# Author’s Response to Peer Reviews of “COVID-19 Pneumonia Diagnosis Using Medical Images: Deep Learning-Based Transfer Learning Approach”

**DOI:** 10.2196/83230

**Published:** 2025-09-26

**Authors:** Anjali Dharmik

**Affiliations:** 1 Royal Holloway University of London, Egham Hill, Egham, TW20 0EX, United Kingdom, 44 7867304854

**Keywords:** computer vision, COVID-19 pneumonia diagnosis, deep learning, transfer learning, medical imaging analysis

*This is the authors’ response to peer-review reports for “COVID-19 Pneumonia Diagnosis Using Medical Images: Deep Learning-Based Transfer Learning Approach.”*

## Round 1 Review

### Reviewer S [1]

#### General Comments

*This paper [* *2* *] focused on the use of artificial intelligence (AI), in particular convolutional neural networks (CNNs) for detection of COVID-19 infections in radiological imaging. The study uses a substantial dataset of over 6000 images, which enhances the reliability of the results and supports robust model training and evaluation. Leveraging well-known CNNs such as VGG16, VGG19, and ResNet-50 demonstrates a practical application of transfer learning, a widely accepted technique in deep learning for medical imaging tasks.*

*However, in the Background and Introduction sections, the authors focused on the importance of rapid and early diagnosis of COVID-19, thus the demand for AI CNNs for diagnosis (“traditional diagnostic methods, such as serologic tests, have limitations, including low sensitivity and longer processing times”), yet this could be achieved easily nowadays with lateral flow devices or rapid antigen tests. Using computed tomography or X-rays to screen COVID-19 is far too expensive and time consuming compared to lateral flow devices or rapid antigen tests.*

*I believe the author was referring to the use of AI CNNs to differentiate COVID-19 pneumonia from other causes of pneumonia. Diagnosis of COVID-19 infection (which is usually mild and self-limiting) is totally different from COVID-19 pneumonia (which might require hospitalization and medical interventions). The authors might consider changing the title of the manuscript to “COVID 19 Pneumonia Diagnosis Analysis Using Transfer Learning–Deep Learning.” Similarly, for section 3.1, “COVID-19 Pneumonia Diagnosis Using Deep Learning” would be more appropriate than “COVID-19 Diagnosis Using Deep Learning.”*

*In addition, the Related Work section is brief and lacks depth. It does not sufficiently review existing medical studies on deep learning for COVID-19 pneumonia diagnosis, making it less comprehensive.*

**Response:** I updated the title to “COVID-19 Pneumonia Diagnosis Using Medical Images: Deep Learning-Based Transfer Learning Approach” and added more details on research into COVID-19 pneumonia diagnosis in the current situation and existing studies.

### Specific Comments

#### Major Comments

1. *Since this is a medical journal, medical terms are encouraged, for example,* anosmia *to replace* loss of smell*;* ageusia *to replace* loss of taste*.*

**Response:** I added more detail about COVID-19 pneumonia diagnosis and the current situation.

*2. Quite a significant number of references were not medical related, but related to AI or computer science. I would suggest the authors visit PubMed to search for more medical-related references. I cannot suggest any particular references to avoid conflicts of interest with certain groups of authors and to avoid self-citation.*

**Response:** I added and updated the references to include medical studies and technical studies.

*3. The author detailed the AI CNN mechanism, yet the features of computed tomography or X-rays that were focused on were not mentioned. Was ground glass appearance the main target, or was it other features like cavitation, extent of lung involvement, or superior location? It would be more valid to evaluate various features targeted by the AI, instead of mentioning how it works.*

**Response:** Thank you for the suggestion. While the study used Grad-CAM to visualize model attention, highlighting features like ground glass opacities and bilateral lower-lobe involvement, we acknowledge that explicitly evaluating a wider range of radiological features (eg, cavitation, extent, and location) would strengthen the clinical validity, and these will be considered in future work.

#### Minor Comments

*4. The author cited many different online references, yet the links or URLs were not available for readers to refer to. I would suggest adding the cited reference sources back for reviewers to assess the appropriateness of the citation, such as references 26 to 28, and for the benefit of readers. For example, reference 9 is not searchable on the internet.*

**Response:** I updated the references.

*5. In section 1.1, “At that point, there have been 98 confirmed cases and no reported deaths in 18 countries outside China...” Please add a reference citation for this factual statement.*

**Response:** I updated this.

*6. In section 1.1, “As of 28 April 2020, 63% of worldwide mortality from the virus was from the Region...” Please clarify the “Region.”*

**Response:** I updated this.

*7. In section 1.3, “Motivation to try to COVID-19 Diagnosis,” the English could be further polished, for example, “Motivation-to-try to COVID-19 Diagnosis” or “Motivation to try towards COVID-19 Diagnosis.”*

**Response:** I updated this.

*8.* Computed tomography*, instead of* computer tomography*, is the proper term in section 3.1.*

**Response:** I updated this.

*9. Abbreviations need not be spelled out again after their first use in the main text. For example, “computer tomography (CT)” in section 3.1 can just be “CT,” since CT has been defined already.*

**Response:** I updated this.

*10. Please be consistent with reference citation formatting; various formats are used in the reference list, for example, “[20] Z. Wu et al Unsupervised feature learning via non-parametric instance discrimination. In Proceedings of the IEEE Conference on Computer Vision and Pattern Recognition, pages 3733‐3742, 2018”; “[24] Md. Islam, F. Karray, R. Alhajj, and J. Zeng. A review on deep learning techniques for the diagnosis of novel coronavirus (covid-19). IEEE Access, vol. 9, pp. 30551‐30572, 2021. doi: 10.1109/ACCESS.2021.3058537”; and “[29] Mohammed K. Hassan, Ali I. El Desouky, Sally M. Elghamrawy, and Amany M. Sarhan. A hybrid real-time remote monitoring framework with nb-woa algorithm for patients with chronic diseases. http://doi.org/10* *.1016/j.future.2018.10.021, 2019. Future Generation Computer Systems, Volume 93, Pages 77‐95, ISSN 0167‐739X.”*

**Response:** I updated this.

*11. To further improve the manuscript, please consider adding figures or tables showing the appearance of COVID-19 versus normal samples. Add to the Limitations section a discussion of potential biases (eg, dataset origin) or generalizability issues (eg, applicability to new variants) to demonstrate critical reflection*

**Response:** I updated this.

### Reviewer AA [3]

#### General Comments

*This manuscript [* *2* *] describes a transfer-learning approach using pretrained convolutional neural networks (VGG16, VGG19, ResNet-50) for binary COVID-19 detection on chest X-ray and computed tomography images. Overall, it tackles a timely problem and reports high accuracy (>97%), but several methodological and reporting issues limit confidence in the findings and their reproducibility.*

#### Specific Comments

##### Major Comments

*1. Lack of clinical validation: no in vivo or clinical ground-truth data are provided. The model’s >97% accuracy is based solely on public datasets; it’s unclear how it performs on real-world, heterogeneous clinical images.*

**Response:** I updated this.

*2. Overfitting and hyperparameter tuning: identical performance across 5 hyperparameter settings for VGG16 suggests under- or overfitting. No learning curves or regularization impact analyses are shown to substantiate robustness claims.*

**Response:** I updated this.

*3. Model comparison baseline: no comparison against simple baselines (eg, logistic regression on hand-crafted features) or recent literature benchmarks is provided, making it difficult to evaluate novelty and real gain.*

**Response:** Thank you for the observation. In our study, we used a baseline AlexNet (a convolutional neural network–inspired architecture) to benchmark performance against more advanced transfer learning models. Our primary focus was on evaluating the effectiveness of various transfer learning approaches.

##### Minor Comments

*4. Repeated headings: “Integration into Mobile/Cloud-based Platform” appears twice in section 1; please consolidate.*

**Response:** I updated this.

*5. Typographical and formatting errors: multiple sentences start without capitalization (eg, “we reviewing to the difference…”) and several references lack publication details (eg, [27,28] list only URLs).*

**Response:** I updated this.

### Reviewer AB [4]

#### General Comments

*This manuscript [* *2* *] investigates the application of deep learning, particularly transfer learning using VGG16, VGG19, and ResNet-50, for diagnosing COVID-19 through computed tomography and X-ray images. The topic is important and timely, especially considering the enduring threat of COVID-19 variants and the burden on global health care systems. The author demonstrates technical familiarity with deep learning techniques, model tuning, and performance evaluation. However, there are areas where the study could be improved to enhance its rigor, clarity, and impact.*

#### Specific Comments

##### Major Comments

*1. Dataset description and bias: the paper mentions using a dataset of 6259 images (4651 COVID-19 cases and 1608 normal cases). However, there is no discussion on potential biases in the dataset, such as the source of the images, demographic diversity (age, gender, and geographic location), or the balance between COVID-19 and normal cases. Addressing these aspects would strengthen the validity of the results. I suggest that the author include a detailed description of the dataset, including sources, demographic information, and steps taken to mitigate bias, and consider discussing the imbalance in the dataset and how it might affect model performance.*

**Response:** I updated this.

*2. Comparative analysis with existing methods: while the paper reports high accuracy (97.73%) for the proposed models, it lacks a comprehensive comparison with other state-of-the-art methods or baseline models. This makes it difficult to assess the novelty and superiority of the proposed approach. I suggest that the author add a comparative table or section that contrasts the performance of VGG16, VGG19, and ResNet-50 with other recent studies or baseline models and highlight the unique contributions of this work.*

**Response:** I updated this.

*3. Clinical relevance and practical deployment: the study focuses on technical performance metrics but does not discuss the clinical applicability of the models. For instance, how would these models integrate into real-world health care settings? What are the potential challenges (eg, computational resources, interpretability for clinicians)? I suggest that the author expand the discussion on clinical relevance, including limitations and practical considerations for deployment in health care systems.*

**Response:** I updated the paper to mention potential challenges, discuss clinical relevance, mention limitations, and discuss practical considerations for deployment in health care systems.

*4. Language and grammar: the manuscript needs extensive language editing. There are frequent grammatical issues, awkward phrasing (eg, “the 1608 belong to healthy people”), and repetition. A professional edit is highly recommended to improve readability and flow.*

**Response:** I updated this.

*5. Figures and tables: several figures (eg, confusion matrices, loss/accuracy curves) are referenced but lack sufficient clarity, labeling, or captions. Figures 4 to 8 must be embedded clearly within the results discussion and interpreted to guide the reader. Ensure figures are high resolution and correctly formatted.*

**Response:** I updated this.

*6. Overstatement of results: the paper claims high performance (97.73% accuracy), yet offers little discussion on external validity or overfitting risks. Since cross-validation was performed on a relatively small dataset, these results may not generalize well. The author should tone down claims and discuss limitations.*

**Response:** I added a detailed discussion of overfitting risks, cross-validation, datasets, and results.

*7. Dataset description and ethics: while the dataset is described as publicly available, the manuscript lacks ethical approval or justification. Clarify whether ethical clearance was required. Also, organize the dataset description into a single, detailed section including data sources, balance between classes, preprocessing applied, and augmentation steps.*

**Response:** I updated the paper to describe collection of the data sources and mention the processing steps.

*8.Evaluation metrics and statistical rigor: the paper heavily relies on accuracy, sensitivity, specificity, and F1-score, but fails to report CIs or conduct statistical tests to validate performance differences between models. Including receiver operating characteristic area under the curve values and visualizations would also strengthen the evaluation.*

**Response:** I included receiver operating characteristic area under the curve values and added a visualization to the Results section.

*9. Novelty and contribution not clearly established: while the paper uses popular convolutional neural network architectures, there is no clear indication of what is novel in this study compared to the extensive body of existing work using these same models on similar datasets. What distinguishes this work? Is it the dataset size, preprocessing technique, tuning strategy, or model ensemble?*

**Response:** I updated these details.

##### Minor Comments

*10. Hyperparameter tuning details: the paper describes hyperparameter tuning but does not explain the rationale behind the selected ranges (eg, learning rate and batch size). A brief justification for these choices would improve reproducibility. I suggest adding a sentence or two explaining why the specified ranges for hyperparameters were chosen.*

**Response:** I added a discussion of the hyperparameter tuning.

*11. Use consistent terminology throughout (eg, “deep learning model” versus “CNN-based model”).*

**Response:** I updated this.

*12. Data augmentation techniques: these are described generically. Specify which augmentations were applied and how frequently. Were augmentation parameters validated?*

**Response:** I updated this discussion with more details.

*13. Please structure the abstract under clear headings, Background, Objective, Methods, Results, and Conclusion, to aid clear reading and comprehension.*

**Response:** I updated this.

## Round 2 Review

### Reviewer S [1]

#### Specific Comments

##### Major Comments

*Some parts of the manuscript[1] used extensive bulleted lists; paragraphs should be used in the manuscript’s main text. If the author deems bullet points more appropriate for the content, the author could format lists as tables.*

**Response:** I rewrote the bullet points as full paragraphs.
